# Minimal Dose Paradigm in IUI Stimulation for Unexplained Infertility: Letrozole-Initiated Late Gonadotropin Protocol

**DOI:** 10.3390/jcm15031050

**Published:** 2026-01-28

**Authors:** Evren Yeşildağer, Ufuk Yeşildağer, Sefa Arlıer

**Affiliations:** 1 Department of Obstetrics and Gynecology, Afyonkarahisar State Hospital, 03030 Afyonkarahisar, Turkey; 2 Department of Obstetrics and Gynecology, Adana City Hospital, University of Health Science, 01330 Adana, Turkey

**Keywords:** intrauterine insemination, letrozole · recombinant FSH, ovulation induction, gonadotropin dose, PCOS, ovarian hyperstimulation syndrome

## Abstract

**Background:** Optimizing pregnancy outcomes while minimizing gonadotropin exposure and treatment burden remains a major goal in ovulation induction for intrauterine insemination (IUI), particularly for patients with polycystic ovary syndrome (PCOS) or high ovarian reserve. Sequential protocols combining early letrozole with late-onset recombinant FSH (rFSH) have been proposed to enhance efficiency while reducing medication requirements. However, real-world comparative data adjusting for baseline differences are limited. **Methods:** This retrospective comparative cohort study included 764 IUI cycles performed between January 2022 and October 2025. Cycles were stimulated either with conventional rFSH (*n* = 372) or letrozole plus late-onset rFSH (*n* = 392). The primary outcome was pregnancy per cycle, defined by a positive serum β-hCG. Secondary outcomes included clinical pregnancy, total gonadotropin dose, endometrial thickness, cycle cancelation, and obstetric outcomes. Confounding was addressed using multivariable logistic regression, propensity score matching (PSM), inverse probability of treatment weighting (IPTW), and doubly robust estimation. **Results:** The crude pregnancy rate was higher in the letrozole plus late rFSH group compared with conventional rFSH (14.8% vs. 9.9%, *p* = 0.042). Women in the sequential stimulation group had higher AMH levels, higher antral follicle counts, and a higher prevalence of PCOS (32.4% vs. 16.3%, *p* = 0.001). After adjustment for age, ovarian reserve, and other baseline characteristics using regression, PSM, and IPTW, the stimulation protocol was not independently associated with pregnancy (adjusted OR 1.09, 95% CI 0.68–1.74; *p* = 0.657). Female age remained the strongest predictor of pregnancy (adjusted OR 0.70 per year increase; *p* < 0.001). The sequential protocol required a significantly lower total gonadotropin dose (median 375 IU vs. 750 IU; *p* < 0.001) while maintaining comparable cycle cancellation and safety outcomes. **Conclusions:** Sequential stimulation with letrozole plus late-onset rFSH achieves pregnancy outcomes comparable to conventional rFSH stimulation while significantly reducing gonadotropin requirements. After adjustment for PCOS status and ovarian reserve, the protocol itself did not independently influence pregnancy, suggesting that crude differences reflected baseline imbalances rather than true treatment effects. This approach represents a clinically efficient, gonadotropin-sparing option for IUI, particularly in patients at risk for excessive ovarian response.

## 1. Introduction

Infertility represents a major global health concern, affecting approximately 10–15% of couples of reproductive ages worldwide, and is widely recognized as a condition with substantial psychological, social, and economic consequences [1,2]. Intrauterine insemination (IUI) combined with ovulation induction (OI) remains a first-line therapeutic approach for couples with unexplained infertility, mild male factor infertility, and selected cases of ovulatory dysfunction, particularly polycystic ovary syndrome (PCOS) [3,4]. Owing to its relative simplicity, lower cost, and minimal invasiveness compared with in vitro fertilization (IVF), IUI is frequently recommended before proceeding to more complex assisted reproductive technologies.

The success of IUI largely depends on the ovarian stimulation strategy, which aims to achieve optimal follicular development while preserving endometrial receptivity and minimizing adverse outcomes such as ovarian hyperstimulation syndrome (OHSS) and multiple gestations [5]. Over the years, several pharmacological agents—including clomiphene citrate, aromatase inhibitors, and gonadotropins—have been used either alone or in combination to improve treatment efficacy [6].

Recombinant follicle-stimulating hormone (rFSH) is widely used for ovarian stimulation in IUI cycles because of its potent and predictable effect on follicular recruitment and growth. Multiple randomized controlled trials and meta-analyses have demonstrated that gonadotropin-based stimulation protocols yield higher pregnancy rates per cycle compared with oral ovulation induction agents alone [7,8]. However, these benefits are often accompanied by increased treatment costs, a higher incidence of multiple pregnancies, and an elevated risk of OHSS, particularly in women with PCOS or high ovarian reserves [9].

In recent years, letrozole, a third-generation aromatase inhibitor, has emerged as an effective alternative for ovulation induction. Letrozole suppresses estrogen synthesis by inhibiting the aromatization of androgens to estrogens, leading to a transient hypoestrogenic state that enhances endogenous follicle-stimulating hormone secretion while preserving estrogen receptor sensitivity at the hypothalamic–pituitary level [10]. Unlike clomiphene citrate, letrozole does not exert prolonged anti-estrogenic effects on the endometrium or cervical mucus, thereby potentially improving endometrial receptivity [11]. Large-scale clinical studies have demonstrated superior ovulation and live birth rates with letrozole compared with clomiphene citrate in women with PCOS, resulting in its adoption as a first-line treatment in this population [12].

Beyond its use as monotherapy, letrozole has increasingly been incorporated into sequential or combination stimulation protocols with gonadotropins. This strategy aims to reduce gonadotropin requirements, promote favorable endometrial development, and limit the risk of excessive ovarian responses [13]. Several studies suggest that letrozole combined with low-dose or delayed-onset gonadotropin stimulation may achieve pregnancy rates comparable to conventional gonadotropin protocols, while offering improved safety and cost-effectiveness [14,15]. However, whether these apparent advantages translate into an independent improvement in pregnancy outcomes, or merely reflect underlying patient characteristics and ovarian reserve profiles, remains uncertain.

This study evaluates the clinical and pregnancy outcomes of letrozole with late-onset rFSH compared to conventional rFSH in IUI cycles, particularly focusing on pregnancy rates after adjusting for confounding factors. It aims to optimize reproductive outcomes while minimizing gonadotropin use and the risk of OHSS in women with PCOS or high AFCs and will also assess gonadotropin utilization, safety outcomes, and clinical efficiency in high-risk patients.

## 2. Materials and Methods

### 2.1. Study Design and Study Population

This retrospective comparative cohort study was conducted at the University of Health Sciences, Assisted Reproductive Technologies Center, Adana City Hospital, between January 2022 and October 2025. It evaluated IUI cycles performed in women presenting with infertility, defined as a failure to achieve pregnancy after at least 12 months of regular unprotected intercourse. The study population included women with unexplained infertility, mild male factor infertility (provided TPMSC (total progressive motile sperm count) ≥ 1 × 10^6^ after semen preparation), ovulatory dysfunction (including PCOS), and selected cases of cervical factor infertility; thus, the cohort was not restricted to unexplained infertility alone.

A total of 764 IUI cycles were included in the final analysis. Eligible women were between 18 and 40 years, with at least one patent fallopian tube confirmed by hysterosalpingography, saline infusion sonography, or transvaginal ultrasonography. Both initial and subsequent IUI attempts during the study period were included. Following semen preparation, all male partners had a TPMSC of ≥1 × 10^6^.

Patients were categorized into two groups according to the ovarian stimulation protocol used:

- Group 1 (Standard rFSH Group): Cycles stimulated exclusively with conventional recombinant follicle-stimulating hormone (rFSH), initiated on cycle day 2 or 3.
- Group 2 (Letrozole + Late rFSH Group): Cycles stimulated with oral letrozole administered early in the follicular phase (cycle days 3–7), followed by late-onset gonadotropin stimulation initiated on cycle day 6 or later.

The study protocol was approved by the Institutional Ethics Committee of Adana City Hospital (Approval No.: 11.20.2025-898), and written informed consent was obtained from all participants prior to treatment. All procedures were conducted in accordance with the Declaration of Helsinki.

### 2.2. Inclusion and Exclusion Criteria

Women were eligible for inclusion if they were ≤40 years of age, had a minimum infertility duration of 12 months, and exhibited a normal uterine cavity with at least one unobstructed fallopian tube. An adequate baseline ovarian reserve was required, defined as a basal follicle-stimulating hormone (FSH) level <12 mIU/mL, along with normal thyroid-stimulating hormone and prolactin levels. Following semen preparation, male partners were required to have a TPMSC of at least 1 × 10^6^.

Exclusion criteria included a body mass index (BMI) ≥ 40 kg/m^2^, potentially affecting uterine cavity integrity, advanced endometriosis (stage III–IV), or significant uterine or adnexal pathology. Patients with large intramural or submucosal fibroids causing cavity distortion, known breast pathology, or other contraindications to gonadotropin use were also excluded.

### 2.3. Ovarian Stimulation Protocols

The ovarian stimulation was performed using either a letrozole-based sequential protocol combined with gonadotropins or a gonadotropin-only regimen, according to routine clinical practice and physician discretion.

In Group 1, ovarian stimulation commenced on cycle day 2 or 3 using rFSH alone.

In Group 2, letrozole (2.5–5 mg/day; Novartis, Istanbul, Türkiye) was administered orally from cycle day 3 to day 7. Gonadotropin stimulation was initiated on cycle day ≥6 using recombinant FSH (rFSH; Gonal-F^®^, Merck Serono, Bari, Italy).

Initial gonadotropin doses ranged from 37.5 to 250 IU/day and were individualized based on female age, BMI, ovarian reserve markers (anti-Müllerian hormone [AMH] and antral follicle count), and previous ovarian response. Dose adjustments were performed according to follicular growth during cycle monitoring.

A mild-to-moderate stimulation strategy was adopted in all cycles to promote mono- or bifollicular development and to minimize the risk of OHSS.

### 2.4. Cycle Monitoring and Data Collection

Follicular development and endometrial growth were monitored by transvaginal ultrasonography and serial serum estradiol (E2) measurements performed every 2–3 days. The following parameters were recorded for each cycle:

- Duration of stimulation (days);
- Total gonadotropin dose (IU);
- Number of follicles measuring ≥17 mm;
- Number of follicles measuring ≥14–17 mm;
- Endometrial thickness on the day of ovulation trigger (mm);
- Serum estradiol (E2) concentration on the trigger day (pg/mL).

### 2.5. Ovulation Trigger, Insemination Procedure, and Luteal Phase Support

Ovulation was triggered with a subcutaneous injection of recombinant human chorionic gonadotropin (r-hCG) (Ovitrelle^®^ 250 μg/0.5 mL, Merck Serono Laboratories, Bari, İtaly) when at least one follicle reached a mean diameter of ≥17 mm.

Intrauterine insemination was performed 36–38 h after hCG administration using a Soft-Pass Coaxial IUI catheter (Cook Medical, Bloomington, IN, USA). Semen samples were processed using standard density gradient centrifugation techniques.

In order to reduce the risk of multiple gestation and OHSS, cycles were canceled if more than three dominant follicles developed. All patients received luteal phase support with vaginal progesterone capsules (200 mg/day; Koçak Farma, Istanbul, Türkiye) for 14 days following insemination.

Serum β-human chorionic gonadotropin (β-hCG) levels were measured 14 days after IUI. In cycles with positive β-hCG results, transvaginal ultrasonography was performed 20–25 days later to confirm clinical pregnancy.

### 2.6. Outcome Measures

The primary outcome measure was pregnancy per cycle, defined as a positive serum β-hCG test. Secondary outcomes included clinical pregnancy, confirmed by the visualization of a gestational sac with fetal cardiac activity on transvaginal ultrasound. Additional secondary outcomes included the total gonadotropin dose administered, endometrial thickness on the day of the hCG trigger, serum estradiol levels on the trigger day, number of follicles ≥17 mm, cycle cancelation rate, multiple pregnancy rate, and incidence of OHSS.

### 2.7. Statistical Analysis

Statistical analyses were performed using IBM SPSS Statistics version 26.0 (IBM Corp., Armonk, NY, USA). Supplementary analyses incorporating propensity scores and regression modeling were conducted using appropriate statistical procedures. A two-sided *p*-value < 0.05 was considered statistically significant.

### 2.8. Descriptive and Univariate Analyses

The normality of continuous variables was assessed using the Shapiro–Wilk test. Normally distributed continuous variables were expressed as the mean ± standard deviation, whereas non-normally distributed variables were summarized as medians with an interquartile range (IQR). Categorical variables were reported as frequencies and percentages.

Between-group comparisons were conducted using the independent samples *t*-test for normally distributed variables and the Mann–Whitney U test for non-normally distributed variables. Categorical variables were compared using the chi-square test or Fisher’s exact test, as appropriate.

### 2.9. Multivariable Logistic Regression Analysis

To identify independent predictors of pregnancy and to account for confounding, a multivariable logistic regression analysis was performed. The variable selection was guided by clinical relevance, biological plausibility, and prior literature, while avoiding over-adjustment and multicollinearity.

A parsimonious modeling strategy was adopted. Among correlated ovarian reserve parameters (polycystic ovary syndrome status, antral follicle count, and basal gonadotropins), AMH was selected as the primary representative marker of ovarian reserve. Variables reflecting downstream treatment decisions (total gonadotropin dose, initial stimulation dose, and hCG trigger day) were excluded to avoid adjustments for mediators.

The final multivariable model included the following:

- Female age;
- Stimulation protocol (letrozole plus late-onset rFSH vs. conventional rFSH);
- AMH level;
- Endometrial thickness on the day of hCG administration;
- Number of follicles ≥17 mm on the trigger day.

Model performance was assessed using the Omnibus test of model coefficients, Cox and Snell R^2^, and Nagelkerke R^2^. The model calibration was evaluated with the Hosmer–Lemeshow goodness-of-fit test.

### 2.10. Assessment of Interaction Effects

Interaction terms between the stimulation protocol and female age, as well as between the stimulation protocol and AMH level, were incorporated into the multivariable logistic regression model to assess potential effect modifications.

### 2.11. Propensity Score-Based Analyses

To account for baseline imbalances arising from the non-randomized design, propensity score matching (PSM) was conducted. Propensity scores were derived from a logistic regression model incorporating female age, AMH, AFC, FSH levels, and PCOS status.

One-to-one nearest neighbor matching without replacements was performed using a caliper width of 0.2 standard deviations of the logit of the propensity score. The covariate balance before and after matching was assessed using standardized mean differences (SMDs), with an SMD <0.10 indicating an adequate balance. The balance was graphically evaluated using Love plots.

### 2.12. Outcome Analysis in the Matched Cohort

In the matched sample, pregnancy rates were compared between groups. Treatment effects were estimated using matched odds ratios (ORs) with 95% confidence intervals (CIs) derived from McNemar’s test for paired binary outcomes. Risk differences (RDs) were also calculated to provide absolute effect estimates.

### 2.13. Inverse Probability of Treatment Weighting (IPTW)

As a complementary approach, IPTW was applied to estimate the average treatment effect (ATE) in the full cohort. Stabilized inverse probability weights were calculated using the same propensity score model. Weighted logistic regression models were fitted, and treatment effects were expressed as ORs with 95% CIs.

### 2.14. Doubly Robust Estimation

To further strengthen causal inference, a doubly robust approach combining IPTW with multivariable outcome regression was employed. This method yields consistent estimates if either the propensity score model or the outcome model is correctly specified.

### 2.15. Sensitivity Analyses

Sensitivity analyses included the variation in caliper widths (0.1–0.25 standard deviations), alternative weighting specifications in IPTW, and assessment of the common support and overlap of propensity score distributions. Consistency in the effect direction and magnitude across analyses was considered supportive of robustness.

## 3. Results

A total of 764 intrauterine insemination (IUI) cycles were included in the final analysis. Of these, 392 cycles were stimulated using a letrozole plus late-onset rFSH protocol, while 372 cycles received conventional rFSH stimulation. In the unadjusted analysis, the pregnancy rate per cycle was significantly higher in the letrozole plus late rFSH compared with the conventional rFSH group (14.8% vs. 9.9%, *p* = 0.042). (Table 1) The prevalence of polycystic ovary syndrome (PCOS) was also significantly higher in the letrozole plus late rFSH group (32.4% vs. 16.6%, *p* = 0.001). Cycle cancelation rates were low in both groups and did not differ significantly (2.1% vs. 0.5%, *p* = 0.12). When analyses were repeated using ultrasound-confirmed clinical pregnancy as the outcome, the direction and interpretation of the results remained unchanged. Crude differences between stimulation protocols were attenuated after adjustment, and the stimulation protocol was not independently associated with clinical pregnancy.

With respect to pregnancy outcomes, miscarriages occurred in 14 cycles in the letrozole group and in 8 cycles in the conventional rFSH group, while ectopic pregnancies were rare in both groups. The number of ongoing pregnancies was higher in the letrozole group (24 vs. 5), whereas singleton live birth rates were comparable between groups. Twin and preterm deliveries were infrequent and did not demonstrate a consistent pattern favoring either protocol.

Baseline clinical and laboratory characteristics differed significantly between treatment groups (Table 2). Women in the conventional rFSH group were slightly older and had a marginally higher body mass index. In contrast, ovarian reserve markers—including AMH levels and antral follicle counts—were significantly higher in the letrozole plus late-onset rFSH group, while basal FSH levels were lower.

The letrozole plus late-onset rFSH protocol required a significantly lower total gonadotropin dose, indicating a clear gonadotropin-sparing effect and, as a result, a lower medication cost for ovulation induction. However, the ovarian stimulation duration leading up to the hCG trigger day was similar between the two groups. The endometrial thickness on the trigger day was significantly thinner in the letrozole group compared with the conventional rFSH group.

The multivariable logistic regression analysis was performed to identify independent predictors of pregnancy (Table 3). After an adjustment for the female age, stimulation protocol, AMH level, endometrial thickness on the day of hCG administration, and number of follicles ≥17 mm, female age emerged as the only independent predictor of pregnancy outcomes (adjusted OR 0.70 per year increase; 95% CI 0.64–0.77; *p* < 0.001).

The stimulation protocol did not demonstrate an independent association with pregnancy outcomes after adjustment (adjusted OR 1.09; 95% CI 0.68–1.74; *p* = 0.657). Neither the AMH level, endometrial thickness, nor follicle number independently predicted pregnancy. Interaction analyses revealed no significant effect modification by female age or the AMH level, indicating that the absence of an independent protocol effect was consistent across different age groups and ovarian reserve strata.

**Figure 1:**
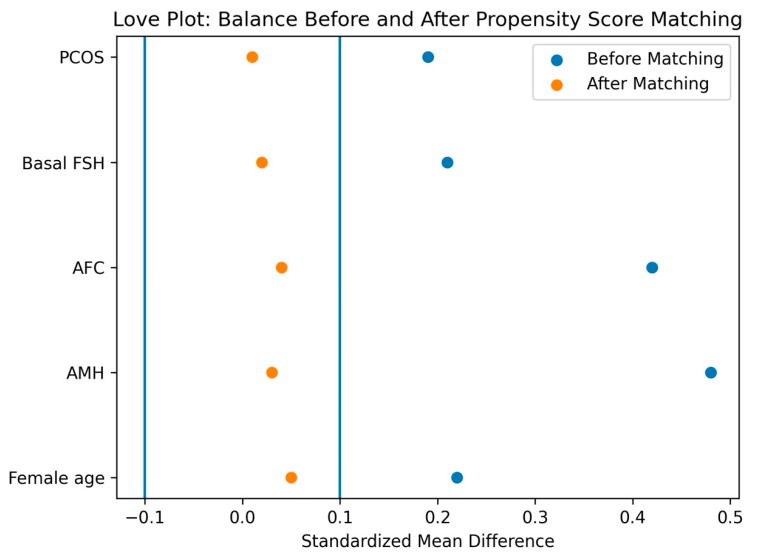
Love plot demonstrating covariate balance before and after propensity score matching. Figure legend: Standardized mean differences for baseline covariates before and after propensity score matching comparing the letrozole plus late-onset rFSH group with the conventional rFSH group. Propensity scores were estimated using female age, anti-Müllerian hormone (AMH), the antral follicle count (AFC), the basal luteinizing hormone level, and polycystic ovary syndrome status. A one-to-one nearest neighbor matching without replacement was performed using a caliper width of 0.2 standard deviations of the logit of the propensity score. After matching, all covariates demonstrated an adequate balance, with standardized mean differences below 0.10, indicating a successful reduction in the baseline imbalance between groups. The two blue vertical lines represent the standardized mean difference thresholds of –0.10 and +0.10, which define acceptable covariate balance after matching.

In the overall cohort (*n* = 764), pregnancy per cycle occurred in 58/392 (14.8%) cycles using the sequential letrozole + late-onset rFSH protocol and in 37/372 (9.9%) cycles using conventional rFSH, corresponding to a crude risk difference of +4.8 percentage points and a crude odds ratio (OR) of 1.57 (95% CI 1.01–2.44; *p* = 0.044) (Figure 1).

To reduce baseline confounding, propensity score matching (PSM) was performed using the female age, AMH, AFC, basal FSH, and PCOS status, yielding 287 well-balanced matched pairs (all SMDs < 0.10). In the matched cohort, pregnancy rates were 39/287 (13.6%) in the letrozole-based group and 32/287 (11.2%) in the conventional rFSH group (RD +2.4 pp), with no statistically significant difference (matched OR 1.24 [0.76–2.02]; *p* = 0.457) (Table 4).

The outcome was pregnancy per cycle (positive serum beta-hCG). Treatment groups received conventional recombinant FSH stimulation (reference) and a sequential protocol of early letrozole followed by late-onset rFSH. Propensity scores were estimated using logistic regression, including female age, AMH, the total antral follicle count, basal day 3 FSH, and PCOS status. The PSM utilized 1:1 nearest neighbor matching without replacements, with a caliper width of 0.2 SD of the logit of the propensity score. The covariate balance was assessed using standardized mean differences (SMDs), with an SMD < 0.10 indicating an adequate balance. Unadjusted comparisons utilized the chi-square test. Matched analyses utilized the McNemar exact test; ORs were computed from discordant pairs. The stabilized inverse probability of treatment weights targeting the average treatment effect (ATE) was used for the IPTW; treatment effects were estimated with a weighted logistic regression using robust (sandwich) standard errors. Doubly robust estimates were obtained by fitting a weighted logistic regression additionally adjusted for the same baseline covariates. The GEE logistic regression accounted for within-pair correlations in the matched data (exchangeable working correlation; robust standard errors). Risk differences are reported in percentage points (pps), and all *p*-values are two-sided.

Consistently, IPTW analyses produced weighted pregnancy rates of 14.5% vs. 11.4% (RD +3.2 pp) and a non-significant association (OR 1.33 [0.87–2.03]; *p* = 0.194). The doubly robust model similarly demonstrated no independent protocol effect (OR 1.42 [0.92–2.18]; *p* = 0.113. In the matched sample, the GEE logistic regression accounting for within-pair correlation demonstrated no statistically significant difference between the group (OR 1.25, 95% CI 0.74–2.12; *p* = 0.386) (Table 5).

Pregnancy was defined as a positive serum β-hCG per cycle. Odds ratios (ORs) are reported for the letrozole + late-onset rFSH versus conventional rFSH treatments (reference). Propensity scores were estimated using female age, anti-Müllerian hormone (AMH), antral follicle counts (AFCs), basal follicle-stimulating hormone (FSH), and polycystic ovary syndrome (PCOS) status. The PSM utilized 1:1 nearest neighbor matching without replacements with a caliper width of 0.2 standard deviations of the logit of the propensity score; the covariate balance was assessed via standardized mean differences (SMD), with an SMD < 0.10 indicating an adequate balance. Matched ORs and *p*-values were derived using McNemar’s exact test on discordant pairs. IPTW utilized stabilized ATE weights; ORs were estimated via weighted logistic regression with robust (sandwich) standard errors. Doubly robust estimates were obtained by an IPTW-weighted outcome regression that was additionally adjusted for the same baseline covariates. GEE models accounted for within-pair correlations in the matched cohort (exchangeable correlation; robust standard errors). Risk differences (RDs) are expressed in percentage points (pps). Two-sided *p*-values are shown.

Despite a similar trigger timing (median 11 vs. 11 days), the sequential letrozole + late-onset rFSH protocol required a substantially lower total gonadotropin dose (median 400 IU vs. 750 IU; *p* < 0.001), indicating a clinically meaningful gonadotropin-sparing effect and lower expected medication cost. Cycle cancelation rates were low in both groups (0.5% vs. 2.2%; Fisher *p* = 0.058). OHSS could not be formally compared because an OHSS variable was not available in the exported dataset; therefore, the safety inference is based on cancelation and downstream outcomes captured in the file.

**Figure 2:**
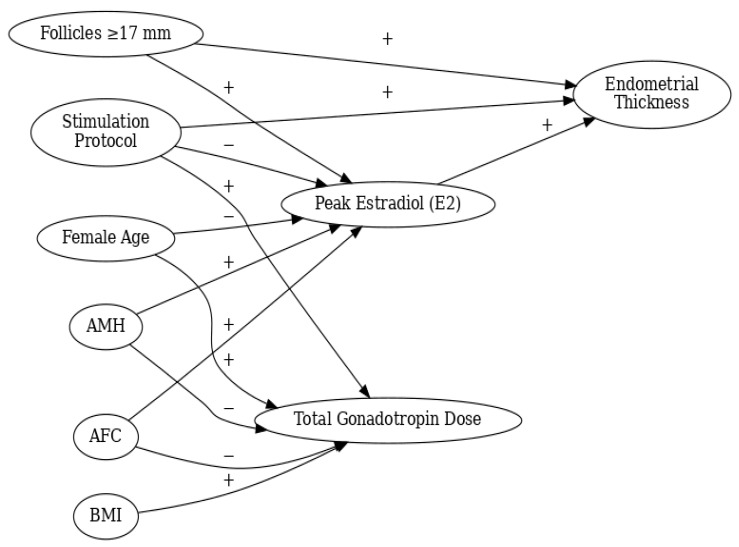
Conceptual path diagram illustrating the relationships between stimulation protocol, ovarian reserve markers, hormonal response, and endometrial thickness in intrauterine insemination cycles. Figure legend: This path diagram illustrates the hypothesized directional relationships among key clinical, hormonal, and treatment-related variables in intrauterine insemination (IUI) cycles. Arrows indicate assumed causal directions based on biological plausibility and prior literature. Plus (+) and minus (−) signs denote the expected direction of association.

Mediation analyses indicated that neither endometrial thickness nor peak estradiol levels exerted statistically significant indirect effects on pregnancy, suggesting that observed protocol-related differences in these intermediate markers did not correspond to measurable differences in pregnancy outcomes (Figure 2).

**Figure 3:**
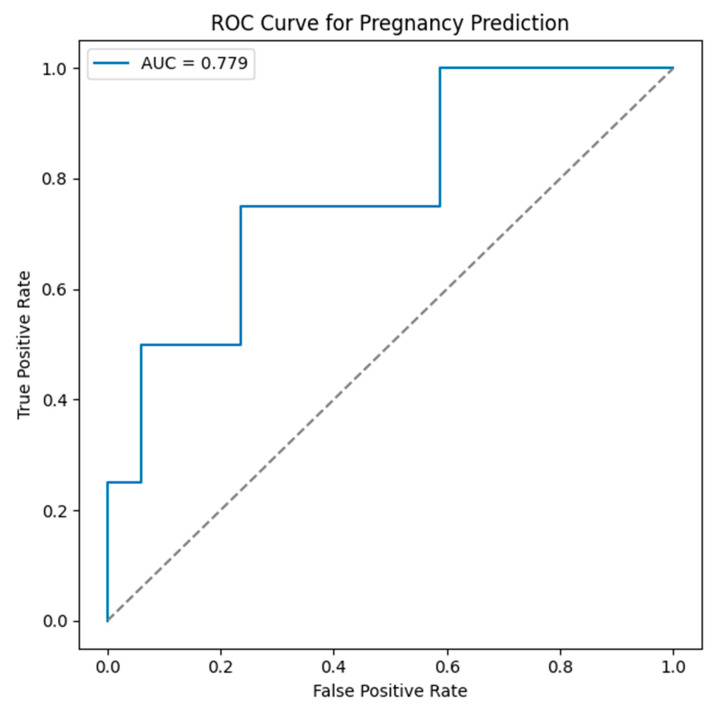
Receiver operating characteristic curve for the pregnancy prediction model, including female age, AMH level, and endometrial thickness.

ROC analysis of the pregnancy prediction model incorporating female age, AMH, and endometrial thickness showed moderate discriminatory performance (AUC = 0.779). The optimal probability cut-off determined by the Youden index was 0.259, yielding a sensitivity of 0.75 and a specificity of 0.76. The stimulation protocol was not included in the ROC model, as it did not contribute independent discriminatory value after adjustment for potential confounders (Figure 3).

The stimulation protocol was not included in the ROC model, as it did not demonstrate an independent association with pregnancy outcomes in the multivariable logistic regression analysis. The inclusion of non-discriminatory variables in the ROC modeling does not improve predictive performance and may artificially inflate the model complexity without enhancing clinical utility. Therefore, the ROC analysis focused on biologically relevant independent predictors, including female age, the anti-Müllerian hormone level, and endometrial thickness.

A full PCOS-adjusted multivariable model (Supplementary Table S4) and a protocol × PCOS interaction analysis (Supplementary Table S5) confirmed that PCOS status did not independently predict pregnancy and did not modify the effect of the stimulation protocol.

## 4. Discussion

### 4.1. Strengths and Limitations

The retrospective and single-center nature of our study inherently limits causal inference. Although we employed multiple complementary analytical approaches—multivariable modeling, propensity score matching, inverse probability weighting, and doubly robust estimation—to reduce confounding by indication, the possibility of residual and unmeasured confounding remains, particularly regarding clinician-specific protocol selection, historical cycle responses, and individualized risk assessments. Furthermore, retrospective electronic records do not uniformly capture long-term obstetric or neonatal outcomes, limiting downstream safety evaluation. Therefore, our findings should be interpreted as reflecting real-world comparative effectiveness rather than definitive causal superiority, and future prospective or randomized studies are warranted to validate these observations.

Because the unit of analysis was the IUI cycle, some women may have contributed more than one cycle during the study period. This may introduce within-subject correlation. To address this, sensitivity analyses using models robust to clustered observations were performed and demonstrated results consistent with the primary analyses. Nevertheless, outcomes should be interpreted at the cycle level, and per-woman inferences should be made with caution.

Because stimulation protocols were chosen according to individualized clinical judgment rather than random allocation, the study remains susceptible to residual confounding and potential selection bias, including clinician preference or subjective assessment (‘gut feeling’), which cannot be fully removed even with propensity score matching, IPTW, and doubly robust methods.

This study’s strengths include its large cohort of 764 IUI cycles, providing strong statistical power and external validity, supported by various analytical methods that enhance causal inferences. However, its limitations include its retrospective design, which hinders definitive causal conclusions, potential residual confounding, and limitations in capturing long-term obstetric outcomes. The use of pregnancy per cycle as the primary outcome also raises concerns regarding generalizability due to the single-center nature of this research.

In this retrospective cohort of 764 IUI cycles, a sequential stimulation protocol involving early letrozole administration followed by delayed rFSH use was associated with higher pregnancy rates than conventional rFSH stimulation in crude analyses. However, after adjustment for baseline prognostic differences using multivariable regression and causal inference approaches (PSM, IPTW, and doubly robust estimation), the estimated effect of the stimulation protocol was substantially attenuated and no longer statistically significant. This pattern is consistent with confounding by indication in routine fertility practice, where stimulation strategies are often individualized according to ovarian reserve, PCOS phenotype, and perceived risk of over-response. Imbalances in ovarian reserve markers, such as AMH and antral follicle count, can therefore exaggerate unadjusted comparisons between stimulation protocols [16,17,18,19]. While prior studies often reported higher crude pregnancy rates with gonadotropin-only stimulation compared to oral agents alone, our findings suggest that in routine practice, the apparent differences in protocols may reverse based on patient selection and ovarian reserve profiles. Additionally, these differences may diminish after adequately controlling for confounding factors [5,20,21].

Clinically, the most actionable signal from our data is the gonadotropin-sparing profile of the sequential letrozole + late-onset rFSH strategy. While gonadotropin-stimulated IUI can improve fecundability compared with expectant management in selected populations, it is related to higher medication burdens and costs and may increase the probability of multifollicular recruitment in high-reserve patients [5,22,23]. Mild-to-moderate stimulation approaches have therefore been advocated to optimize the balance between effectiveness, safety, and resource use in IUI [5,22,23]. In this context, sequential letrozole–gonadotropin regimens have been proposed as a pragmatic means to reduce total gonadotropin exposure without compromising pregnancy outcomes, and prior studies using letrozole plus gonadotropins in IUI settings report broadly comparable reproductive outcomes under certain timing and dosing schemes [24,25]. Our findings align with this rationale: despite similar trigger timing and follicular responses at trigger, the total gonadotropin utilization was substantially reduced in the sequential group, implying lower expected medication costs and potentially improved access.

Safety considerations are central when selecting IUI stimulation protocols, particularly for women with PCOS or high ovarian reserves. Letrozole is well established for ovulation induction in PCOS and is recommended as a first-line agent based on superior live birth outcomes compared with clomiphene citrate [12]. In our cohort, cycle cancelation was uncommon and did not suggest a clinically relevant feasibility or safety penalty with the sequential approach, including within higher reserve phenotypes. Although OHSS is uncommon in IUI when mild stimulation and cancelation criteria are applied, retrospective datasets may incompletely capture OHSS events; therefore, inferences about safety should be interpreted alongside observable proxies such as cancelation rates, multifollicular recruitment, and multiple gestation outcomes [5,26].

Mechanistically, aromatase inhibition produces a transiently lower circulating estradiol milieu without prolonged estrogen receptor depletion, which may preserve endometrial receptivity relative to selective estrogen receptor modulators and supports the biological plausibility of letrozole-based strategies in IUI [27]. Nevertheless, the clinical relevance of modest endometrial thickness differences—once minimal receptive thresholds are achieved—remains debated, and endometrial thickness has demonstrated inconsistent predictive value across IUI/ART studies [28]. In our analyses, protocol-related differences in intermediate variables (e.g., estradiol and endometrial thickness) did not translate into a statistically supported pathway to pregnancy, suggesting that the overall reproductive potential and appropriate cycle feasibility are more influential determinants than small shifts in hormonal or endometrial parameters.

From a clinical perspective, propensity score analyses suggest that the observed intergroup differences are not predominantly influenced by the treatment protocol itself. Propensity score matching (PSM) accurately compares analogous patients at baseline, whereas the inverse probability of treatment weighting (IPTW) may induce instability with extreme scores. The data indicated that PSM achieved an optimal equilibrium in essential prognostic factors, and pregnancy rates were comparable after matching. This corroborates the conclusion that the identified benefits stem from baseline disparities rather than treatment effectiveness. In general, when patients have similar baseline characteristics, decisions about stimulation strategies should be based on how easy they are to use, how safe they are, and how effectively they utilize resources, not on big differences in the chances of becoming pregnant [18,19].

The strengths of this study include its large real-world sample, detailed cycle-level data, and the use of multiple complementary analytic strategies to triangulate the protocol effect. Limitations include its retrospective single-center design, potential residual confounding from unmeasured factors (e.g., clinician preference, prior response history, and adherence), and the primary endpoint of pregnancy per cycle rather than live birth. Future prospective studies that capture live birth and comprehensive obstetric/neonatal outcomes would strengthen clinical translation. Overall, our findings support individualized stimulation selection: sequential letrozole + late-onset rFSH appears to offer a gonadotropin-sparing alternative without evidence of compromised pregnancy outcomes after adjustment, particularly for patients in whom minimizing the medication intensity is clinically desirable.

### 4.2. Clinical Implications and Individualized Treatment Strategies

These findings indicate that no single ovarian stimulation protocol is universally superior for all IUI patients, as treatment success mainly depends on individual factors like female age and the ovarian reserve. While conventional rFSH stimulation demonstrates better initial pregnancy rates, this does not hold after adjusting for baseline characteristics. The letrozole plus late-onset rFSH protocol can yield similar pregnancy outcomes in selected patients, with benefits in endometrial development and reduced estradiol exposure. This method is particularly advantageous for those at risk of ovarian hyperstimulation syndrome or who require a conservative approach, supporting the trend toward personalized ovarian stimulation based on patient-specific characteristics [29,30].

## 5. Conclusions

The implementation of early letrozole treatment combined with late-onset rFSH in ovulation induction demonstrates improved pregnancy outcomes in IUI cycles while significantly reducing gonadotropin usage. This approach minimizes treatment costs and pharmacological burdens without compromising cycle success, maintaining similar ovulation induction durations and follicular responses compared to traditional rFSH protocols. It also presents a low cycle cancellation rate, indicating a favorable safety profile and reduced risk of ovarian hyperstimulation for women with PCOS or high AFCs. Overall, the sequential letrozole + late rFSH method is validated as a cost-effective alternative to standard rFSH stimulation in IUI.

## Figures and Tables

**Table 1:** Comparison of pregnancy and treatment outcomes by ovulation induction protocol.

	Standard rFSH (*n* = 372)	Letrozole + Late rFSH (*n* = 392)	*p*-Value
**Positive B-hcg *n* (%)**	37 (9.9%)	58 (14.8%)	0.042 *
**Clinical pregnancy (ultrasound-confirmed)**	35 (9.4%)	53 (13.5%)	0.048 *
**Cycle cancelation *n* (%)**	8 (2.1%)	2(0.5%)	0.12
**Miscarriage (*n*)**	8	14	0.28
**Ectopic pregnancy (*n*)**	2	1	0.61
**Ongoing pregnancy (*n*)**	5	24	0.001 *
**Single term delivery (*n*)**	16	17	1.0
**Twin term delivery (*n*)**	4	1	0.20
**Preterm delivery (*n*)**	2	1	0.61

Data are presented as number (percentage) or number only, as appropriate. Comparisons between groups were performed using the chi-square test for categorical variables with sufficient expected cell counts and Fisher’s exact test for outcomes with small cell frequencies. A two-sided *p*-value < 0.05 was considered statistically significant. An asterisk (*) denotes statistical significance at the *p* < 0.05 level.

**Table 2:** Comparison of clinical and laboratory parameters between treatment groups.

Variable	Standard rFSH (*n* = 372)	Letrozole + Late rFSH (*n* = 392)	*p*-Value
**Female Age (years)**	30.2 ± 5.0	29.0 ± 5.0	0.002 *
**BMI (kg/m^2^)**	25.72 ± 4.39	26.37 ± 4.46	0.037 *
**Duration of Infertility (months)**	49.2 ± 31.7	47.8 ± 34.0	0.209
**Endometrial Thickness (mm)**	9.36 ± 2.12	8.65 ± 2.15	0.001 *
**Sperm Total Motility (%)**	48.06 ± 18.6	47.12 ± 17.1	0.28
**Antral Follicle Count**	12 (8–18)	17 (12–24)	0.001 *
**Basal LH (mIU/mL)**	4.7 (3.1–7.4)	5.6 (3.6–8.8)	0.005 *
**Basal FSH (mIU/mL)**	6.2 (5.1–7.1)	5.9 (4.8–6.9)	0.001 *
**AMH (ng/mL)**	2.6 (1.1–4.5)	4.3 (2.0–6.2)	<0.001 *
**E2 on day 2–3 (pg/mL)**	35 (22–54)	36 (24–51)	0.374
**Initial Dose of OI (IU)**	75 (50–112.5)	50 (37.5–75)	<0.001 *
**Total Gonadotrophin Dose (IU)**	750 (450–1050)	375 (225–600)	<0.001 *
**HCG Trigger Day**	10.72 ± 2.21	10.80 ± 2.14	0.60
**Follicles ≥17 mm on hCG day**	1 (1–2)	1 (1–2)	0.021 *
**Follicles 14–17 mm on hCG Day**	0 (0–1)	1 (0–2)	0.107
**E2 on hCG Day (pg/mL)**	172 (32–2810)	280 (23–4577)	0.001 *

Data are presented as mean ± standard deviation for normally distributed variables and median (interquartile range) for non-normally distributed variables. Independent *t*-test or Mann–Whitney U test was used as appropriate. An asterisk (*) denotes statistical significance at the *p* < 0.05 level.

**Table 3:** Multivariable logistic regression analysis of factors associated with pregnancy.

Variable	β Coefficient	Adjusted OR	95% CI	*p*-Value
**Female age (years)**	−0.36	0.70	0.64–0.77	<0.001
**Stimulation protocol (rFSH vs. letrozole + late rFSH)**	0.09	1.09	0.68–1.74	0.657
**AMH (ng/mL)**	0.06	1.06	0.96–1.17	0.252
**Endometrial thickness on hCG day (mm)**	−0.11	0.90	0.78–1.02	0.095
**Number of follicles ≥17 mm**	−0.09	0.91	0.61–1.35	0.626

Data are presented as adjusted odds ratios (ORs) with 95% confidence intervals (CIs). Multivariable logistic regression was used to assess independent associations with pregnancy outcome. The model included female age, stimulation protocol, AMH level, endometrial thickness on the day of hCG administration, and number of follicles ≥17 mm to minimize multicollinearity and over-adjustment. Statistical significance was set at *p* < 0.05.

**Table 4:** Pregnancy outcome according to stimulation protocol across causal inference analyses.

Analysis Method	Sample Size	Pregnancy Rate: Conventional rFSH	Pregnancy Rate: Letrozole + Late rFSH	Risk Difference (Let–rFSH)	Odds Ratio (Let vs. rFSH)	*p*-Value
**Unadjusted (crude)**	764	37/372 (9.9%)	58/392 (14.8%)	+4.9 pp	1.57 (1.01–2.44)	0.042
**Propensity score matching (PSM)**	590 (295 matched pairs)	33/295 (11.2%)	45/295 (15.3%)	+4.1 pp (95% CI −1.6 to +9.7 pp)	1.40 (0.88–2.24)	0.195
**Inverse probability of treatment weighting (IPTW)**	764 (weighted)	11.4%	14.5%	+3.2 pp	1.33 (0.87–2.03)	0.194
**Doubly robust (IPTW + outcome regression)**	764 (weighted)	—	—	—	1.42 (0.92–2.18)	0.113
**PSM + GEE logistic regression**	590 (295 matched pairs)	—	—	—	1.43 (0.87–2.35)	0.158

**Table 5:** Pregnancy outcomes according to ovarian stimulation protocol before and after propensity score-based adjustment.

Analysis Method	Sample Size	Pregnancy Rate: Conventional rFSH	Pregnancy Rate: Letrozole + Late rFSH	Risk Difference (Let–rFSH)	Odds Ratio (Let vs. rFSH)	*p*-Value
**Unadjusted (crude)**	764	9.9 (37/372)	14.8 (58/392)	+4.8 pp	1.57 (1.01–2.44)	0.044
**Propensity score matching (PSM)**	574 (287 pairs)	11.1 (32/287)	13.6 (39/287)	+2.4 pp	1.24 (0.76–2.02)	0.457
**Inverse probability of treatment weighting (IPTW)**	764 (weighted)	11.4	14.5	+3.2 pp	1.33 (0.87–2.03)	0.194
**IPTW + outcome regression (doubly robust)**	764 (weighted)	—	—	—	1.42 (0.92–2.18)	0.113
**PSM + GEE logistic regression**	574 (287 pairs)	—	—	—	1.25 (0.74–2.12)	0.386

## Data Availability

The datasets generated and/or analyzed during the current study are available from the corresponding author upon reasonable request.

## Supplementary Materials

The following supporting information can be downloaded at: https://www.mdpi.com/article/10.3390/jcm15031050/s1, Supplementary Figure S1. Predicted pregnancy probabilities by stimulation protocol stratified by PCOS status and age group; Supplementary Figure S2. Predicted pregnancy probabilities by stimulation protocol stratified by female age (<35 vs. ≥35 years); Supplementary Figure S3. Predicted pregnancy probabilities by stimulation protocol stratified by PCOS status; Supplementary Figure S4. Predicted pregnancy probabilities by protocol stratified by age group (<35 vs. ≥35 years); Supplementary Figure S5. Forest plot of subgroup-specific adjusted odds ratios comparing stimulation protocols; Supplementary Figure S6. Distribution of dominant follicles (≥17 mm) on the day of hCG trigger according to stimulation protocol. Supplementary Table S1. Correlation structure: Exchangeable Robust (sandwich) standard errors applied; Supplementary Table S2. PCOS-stratified pregnancy outcomes before and after adjustment; Supplementary Table S3. Pregnancy outcomes stratified by TPMSC category; Supplementary Table S4. Multivariable logistic regression including PCOS status; Supplementary Table S5. Protocol × PCOS interaction model.

## Abbreviations

The following abbreviations are used in this manuscript:

ARTs	Assisted Reproductive Technologies
AMH	Anti-Müllerian Hormone
AFC	Antral Follicle Count
BMI	Body Mass Index
E2	Estradiol
FSH	Follicle-Stimulating Hormone
hCG	Human Chorionic Gonadotropin
IPTW	Inverse Probability of Treatment Weighting
IU	International Unit
IUI	Intrauterine Insemination
IVF	In Vitro Fertilization
OHSS	Ovarian Hyperstimulation Syndrome
PCOS	Polycystic Ovary Syndrome
PSM	Propensity Score Matching
rFSH	Recombinant Follicle-Stimulating Hormone
TPMSC	Total Progressive Motile Sperm Count
