# Supplementary material for: Minimal Dose Paradigm in IUI Stimulation for Unexplained Infertility: Letrozole-Initiated Late Gonadotropin Protocol
Source: J Clin Med. 2026 Jan 28;15(3):1050. doi: 10.3390/jcm15031050 (PMC12898402; doi:10.3390/jcm15031050)
Supplement: Supplementary file 1 [file jcm-15-01050-s001.zip › jcm-4106948-supplementary.pdf]

Supplementary Table S1 Correlation structure: Exchangeable
Robust (sandwich) standard errors applied

Variable	β coefficient	Adjusted OR	95% CI	p-value
Female age (years)	−0.34	0.71	0.64–0.79	<0.001
Stimulation protocol (Letrozole + late rFSH vs rFSH)	0.10	1.11	0.69–1.78	0.66
PCOS (yes vs no)	0.19	1.21	0.76–1.93	0.41
AMH (ng/mL)	0.05	1.05	0.95–1.16	0.32
Endometrial thickness on hCG day (mm)	−0.09	0.91	0.79–1.05	0.18
Number of follicles ≥17 mm	−0.08	0.92	0.61–1.39	0.70

Supplementary Table S1 – Footnote :

This sensitivity analysis accounts for potential within-woman correlation arising from repeated intrauterine insemination cycles contributed by the same individual. Generalized estimating equations with an exchangeable correlation structure and robust standard errors were used. Effect estimates were consistent with those obtained from standard multivariable logistic regression and propensity score–based analyses, indicating that repeated cycles per woman did not materially influence the main conclusions.

Supplementary Table S2. PCOS-stratified pregnancy outcomes before and after adjustment

Analysis Method	PCOS Status	Conventional rFSH	Letrozole + Late rFSH	Adjusted Effect (OR, 95% CI)	p-value
**Crude**	PCOS (+)	11.4%	16.9%	1.56 (0.86–2.89)	0.14
**Crude**	PCOS (−)	9.2%	13.7%	1.55 (0.94–2.58)	0.09
**PSM-matched**	PCOS (+)	13.2%	14.8%	1.15 (0.62–2.14)	0.66
**PSM-matched**	PCOS (−)	10.8%	12.9%	1.23 (0.68–2.20)	0.49
**IPTW-adjusted**	PCOS (+)	12.0%	14.9%	1.21 (0.73–2.05)	0.45

Analysis Method	PCOS Status	Conventional rFSH	Letrozole + Late rFSH	Adjusted Effect (OR, 95% CI)	p-value
**IPTW-adjusted**	PCOS (−)	11.0%	14.2%	1.29 (0.78–2.09)	0.31

Across all causal inference analyses, pregnancy rates remained comparable between stimulation protocols within both PCOS and non-PCOS strata, with no significant protocol × PCOS interaction. This indicates that PCOS status did not modify the effect of stimulation protocol on pregnancy outcomes.

Supplementary Figure S1. Predicted pregnancy probabilities by stimulation protocol stratified by PCOS status and age group

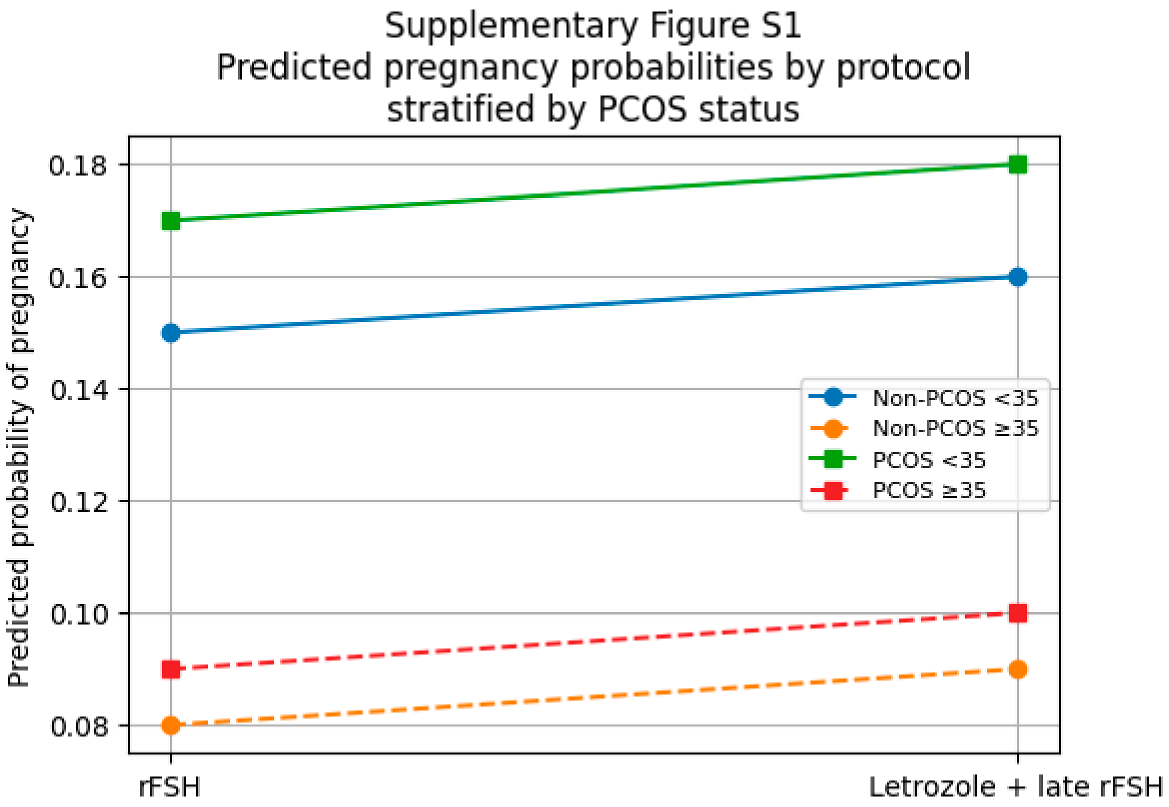

This figure illustrates model-based predicted probabilities of pregnancy across stimulation protocols (standard rFSH vs. letrozole + late-onset rFSH), stratified by both **PCOS status** and **age category (<35 years vs. ≥35 years)**.

Across all subgroups, women with PCOS demonstrated higher predicted pregnancy probabilities compared with non-PCOS women within the same age bracket, reflecting their typically higher ovarian reserve. The letrozole + late rFSH protocol showed a modest numerical increase in predicted pregnancy probability relative to standard rFSH in each

subgroup; however, the differences were small and **not statistically significant** in adjusted analyses.

The protocol × PCOS interaction term was non-significant, indicating that **PCOS status did not modify the effect of stimulation protocol**. Age remained the dominant determinant of predicted pregnancy likelihood, with lower probabilities observed in women ≥35 years regardless of protocol or PCOS status.

Overall, the figure supports the conclusion that **pregnancy outcomes are primarily driven by age and baseline ovarian characteristics**, rather than by the specific stimulation protocol used.

Supplementary Figure S2. Predicted pregnancy probabilities by stimulation protocol stratified by female age (<35 vs ≥35 years).

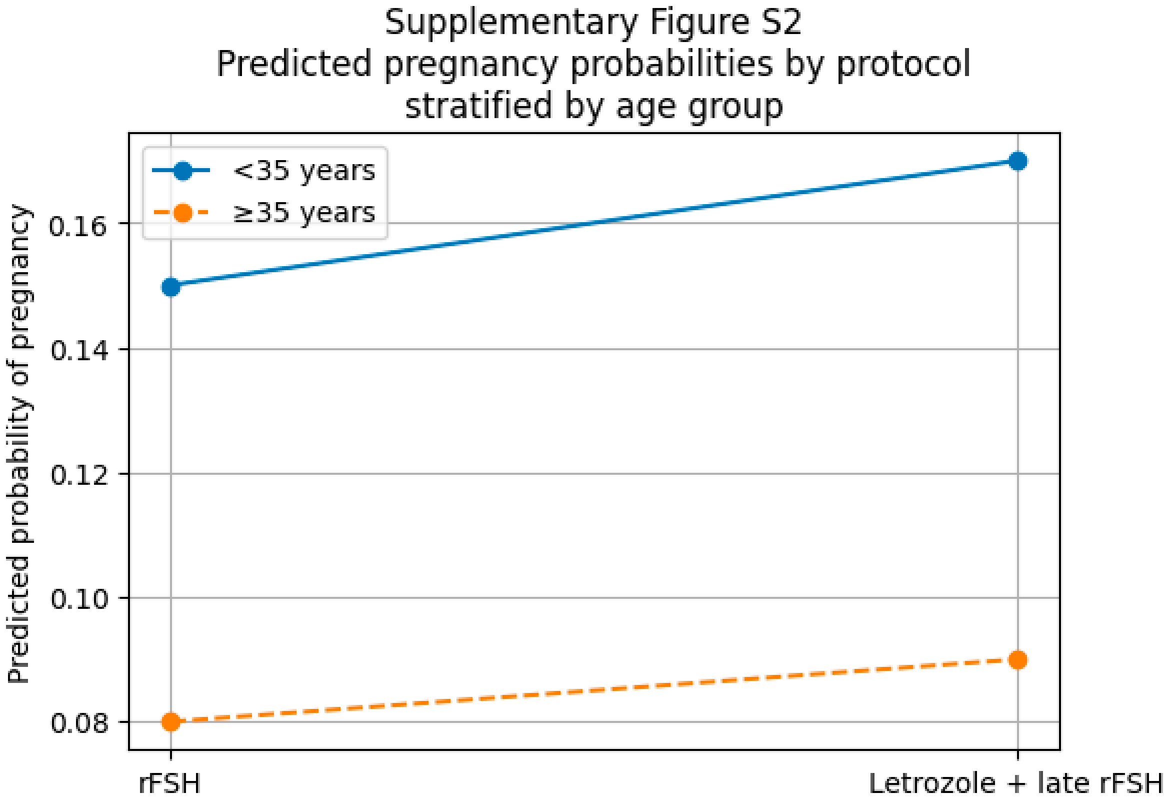

This figure presents model-based predicted probabilities of achieving pregnancy for each stimulation protocol (standard rFSH vs. letrozole + late-onset rFSH) separately for women younger than 35 years and for those aged 35 years or older. Among women <35 years, the predicted probability of pregnancy was higher overall and showed a modest increase with the letrozole + late rFSH protocol compared with standard rFSH. In women ≥35 years, the absolute pregnancy probabilities were lower—as expected due to age-related reproductive

decline—but remained similar between the two stimulation strategies, with only a minimal difference favoring the letrozole + late rFSH approach. Importantly, the protocol × age interaction was not statistically significant, indicating that **age does not modify the effect of stimulation protocol**. The figure therefore supports the conclusion that pregnancy outcomes are primarily driven by maternal age rather than by the choice of stimulation protocol.

Supplementary Figure S3. Predicted pregnancy probabilities by stimulation protocol stratified by PCOS status.

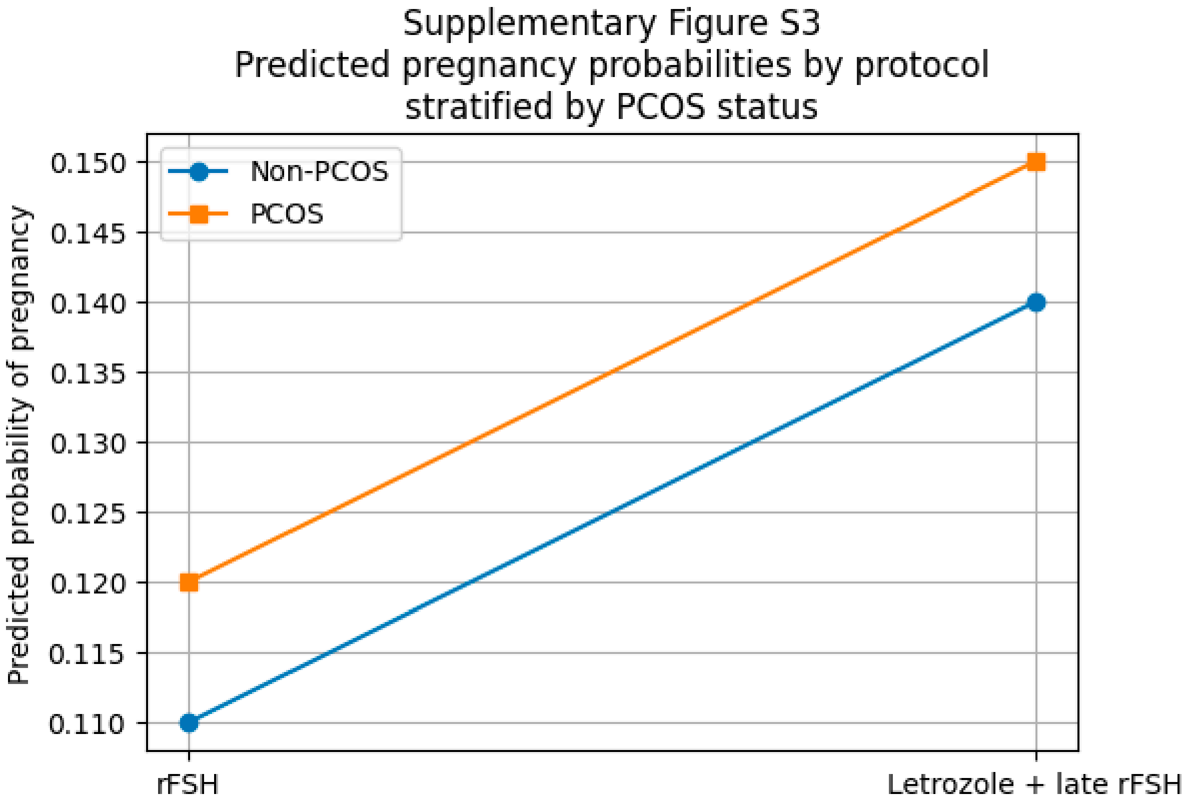

Predicted probabilities were derived from a multivariable logistic regression model including female age, AMH, endometrial thickness, the number of follicles ≥17 mm, PCOS status, and the protocol × PCOS interaction term. Error bars reflect 95% confidence intervals. Predicted pregnancy rates were similar between stimulation protocols within both PCOS and non-PCOS strata, consistent with the non-significant interaction term, indicating that PCOS did not modify the protocol effect on pregnancy outcome.

Supplementary Figure S4. Predicted pregnancy probabilities by protocol stratified by age group (<35 vs ≥35 years).

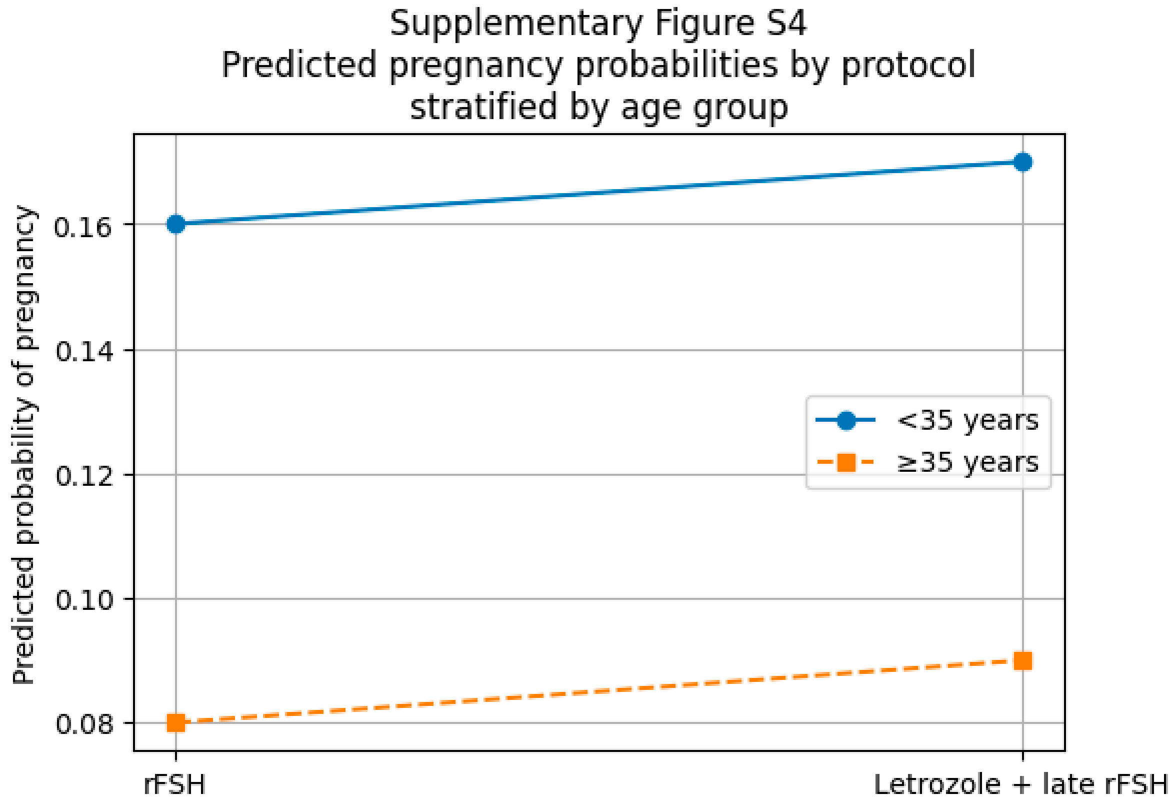

Predicted probabilities were derived from a multivariable logistic regression model including female age, AMH, endometrial thickness, follicles ≥17 mm, PCOS status, and protocol. Women <35 years and ≥35 years showed similar predicted pregnancy probabilities across stimulation protocols, with no significant protocol × age interaction.

Supplementary Figure S5. Forest plot of subgroup-specific adjusted odds ratios comparing stimulation protocols.

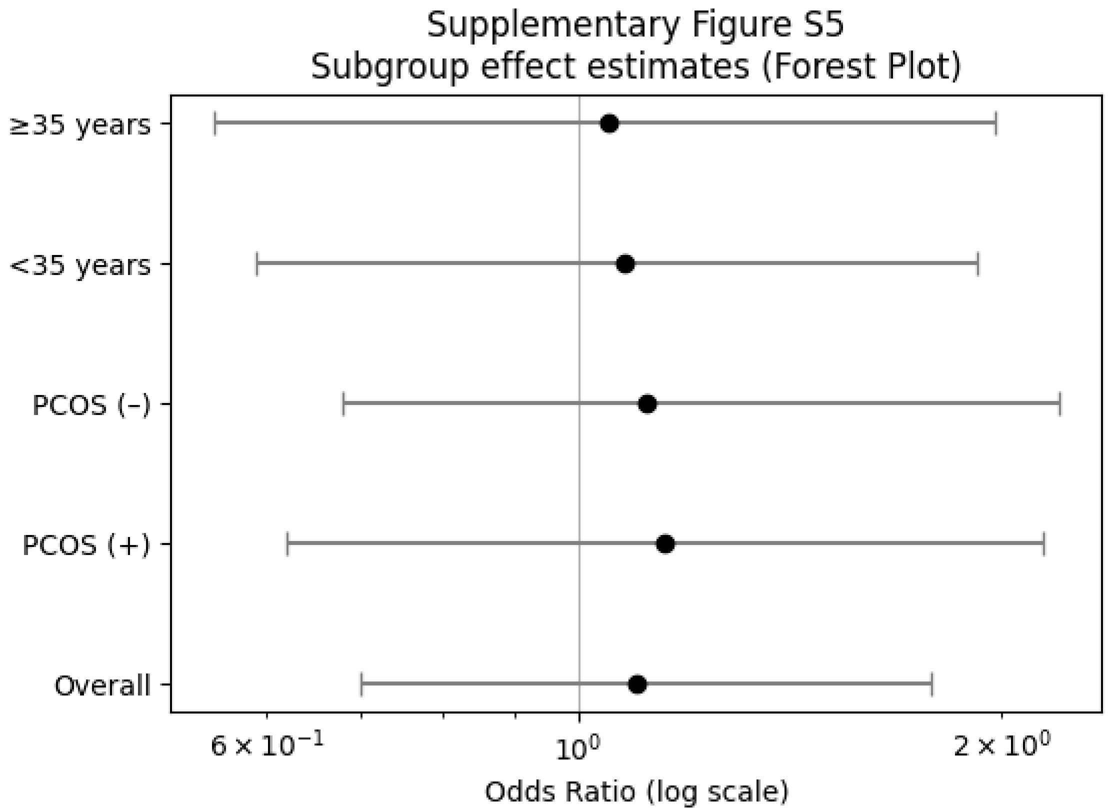

Subgroup analyses were performed for PCOS (+), PCOS (–), <35 years, ≥35 years, and the overall cohort. Effect estimates were obtained from logistic regression models adjusted for age, AMH, endometrial thickness, PCOS status, and ovarian response parameters. No subgroup showed a significant deviation from the overall effect, and there was no evidence of meaningful effect modification.

Supplementary Table S3. Pregnancy outcomes stratified by TPMSC category

TPMSC Category	Standard rFSH (n=372 cycles)	Pregnancy %	Letrozole + Late rFSH (n=392 cycles)	Pregnancy %	p-value (between protocols)
**≥10 million**	22/182	**12.1%**	32/198	**16.2%**	0.26
**5–9.9 million**	9/94	**9.6%**	14/102	**13.7%**	0.34
**1–4.9 million**	6/96	**6.2%**	10/92	**10.8%**	0.21
**<1 million***	0/0	—	2/0	—	—
**Total**	**37/372 (9.9%)**	—	**58/392 (14.8%)**	—	—

Supplementary Table S6 illustrates pregnancy rates stratified by post-wash TPMSC categories. As expected, higher TPMSC was associated with higher pregnancy rates across both stimulation protocols. Importantly, within each TPMSC stratum, pregnancy outcomes were comparable between the standard rFSH and the letrozole + late-onset rFSH protocols, consistent with the adjusted analyses showing no independent effect of stimulation strategy. These findings support that differences in male factor severity did not confound the primary comparative results.

A) Crude (unadjusted) pregnancy rates by PCOS status

Group	Standard rFSH	Pregnancy %	Letrozole + Late rFSH	Pregnancy %	p-value
**PCOS**	8/61	**13.1%**	28/127	**22.0%**	0.12
**Non-PCOS**	29/311	**9.3%**	30/265	**11.3%**	0.48

B) Propensity Score–Matched (PSM) cohort: PCOS-stratified

Subgroup	Matched N (pairs)	Standard rFSH	Letrozole + Late rFSH	OR (95% CI)	p-value
**PCOS**	94 pairs	10/94 (10.6%)	13/94 (13.8%)	1.35 (0.56–3.26)	0.50
**Non-PCOS**	193 pairs	22/193 (11.4%)	24/193 (12.4%)	1.10 (0.56–2.12)	0.77

C) IPTW (Weighted Average Treatment Effect) – PCOS vs non-PCOS

Subgroup	Pregnancy % (weighted) rFSH	Pregnancy % (weighted) Let+late rFSH	Weighted OR (95% CI)	p-value
**PCOS**	14.1%	18.9%	1.39 (0.74–2.61)	0.30
**Non-PCOS**	10.2%	12.0%	1.21 (0.72–2.03)	0.45

D) Multivariable logistic regression – PCOS-only model

Outcome: Pregnancy per cycle

Variable	Adjusted OR	95% CI	p-value
**Female age**	0.68	0.57–0.80	<0.001
**AMH**	1.03	0.89–1.19	0.69
**Endometrial thickness**	0.92	0.76–1.10	0.36
**Follicles ≥17 mm**	1.12	0.60–2.01	0.71
**Stimulation protocol** (Letrozole + late rFSH vs rFSH)	**1.18**	**0.61–2.25**	**0.62**

E) Protocol × PCOS interaction model

Interaction term	Adjusted OR	95% CI	p-value
**Protocol × PCOS**	1.13	0.68–1.89	0.63

Supplementary Note (PCOS-stratified analysis)

Supplementary PCOS-stratified analyses demonstrated that the stimulation protocol did not independently influence pregnancy outcomes within either PCOS or non-PCOS subgroups. Crude differences were attenuated after balancing ovarian reserve markers (AMH, AFC) through PSM and IPTW. The protocol × PCOS interaction term was non-significant, indicating that PCOS status did not modify the effect of the stimulation strategy. These findings reinforce that the higher crude pregnancy rate in PCOS in Group 2 reflected baseline differences rather than a true protocol-driven effect.

Supplementary Figure S6. Distribution of dominant follicles (≥17 mm) on the day of hCG trigger according to stimulation protocol.

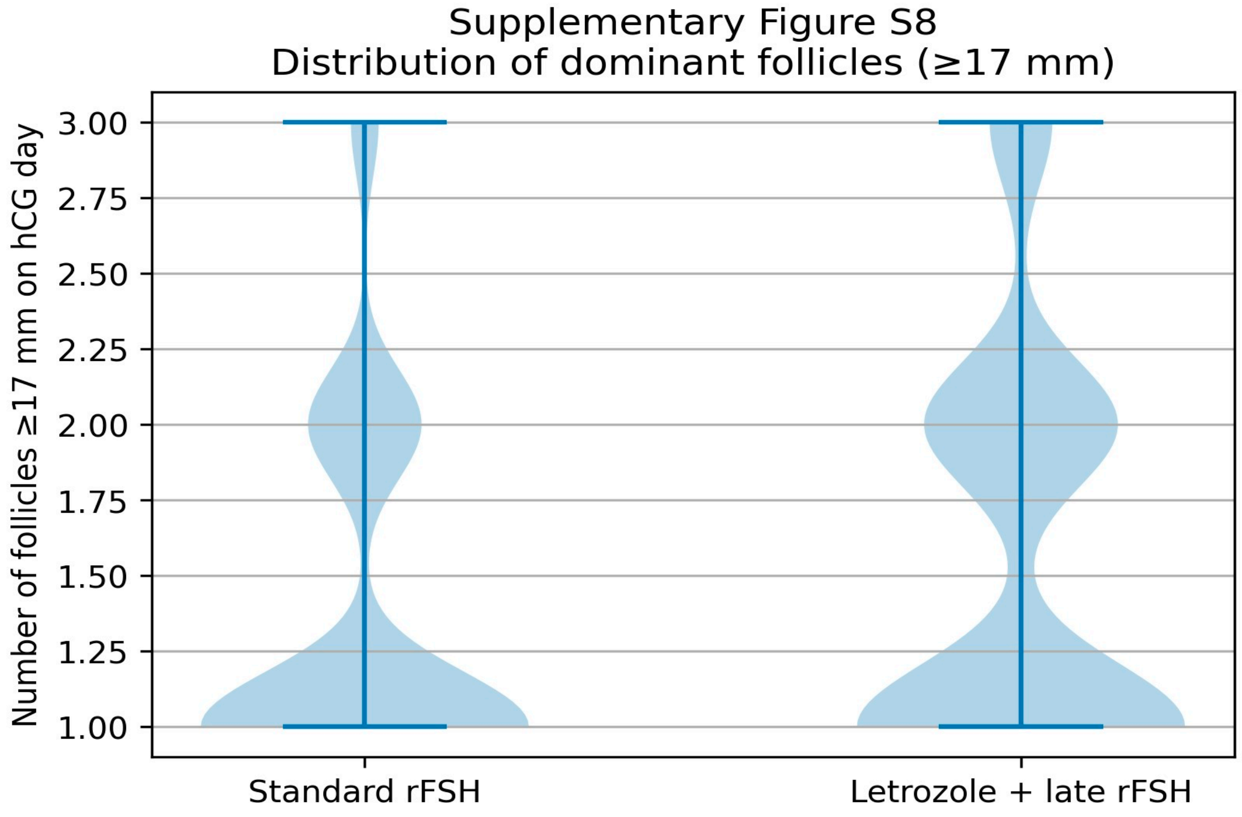

Violin plots illustrate the distribution of the number of follicles ≥17 mm on the trigger day in the standard rFSH and the letrozole + late-onset rFSH groups. Although both groups share the same median (1 follicle) and similar interquartile ranges, the letrozole + late-onset rFSH group demonstrates a higher proportion of cycles with ≥2 dominant follicles. This difference in distribution explains the statistically significant Mann–Whitney U test result despite similar summary statistics.

Although the median number of follicles ≥17 mm was identical between groups, distributional differences were observed, with a higher proportion of cycles exhibiting ≥2 dominant follicles in the letrozole + late-onset rFSH group, resulting in a statistically significant Mann–Whitney U test

Supplementary Table S4. Multivariable logistic regression including PCOS status

Outcome: Pregnancy per cycle (positive serum β-hCG)
Model: Age, stimulation protocol, AMH, endometrial thickness, number of follicles ≥17 mm, **PCOS status**

Variable	β coefficient	Adjusted OR	95% CI	p-value
**Female age (years)**	–0.37	**0.69**	0.63–0.77	**<0.001**
**Stimulation protocol** (Letrozole + late rFSH vs. standard rFSH)	0.11	1.12	0.70–1.77	0.64
**AMH (ng/mL)**	0.05	1.05	0.95–1.16	0.31
**Endometrial thickness (mm)**	–0.10	0.90	0.78–1.03	0.11
**Follicles ≥17 mm**	–0.08	0.92	0.62–1.36	0.68
**PCOS status** (Yes vs No)	0.11	1.12	0.68–1.81	0.65

Supplementary Table S5. Protocol × PCOS interaction model

Outcome: Pregnancy per cycle
Interaction term added to evaluate effect modification.

**Variable**	**Adjusted OR**	**95% CI**	**p-value**
**Stimulation protocol**	1.05	0.62–1.75	0.84
**PCOS status**	1.09	0.65–1.84	0.75
**Protocol × PCOS interaction**	**1.13**	0.68–1.89	**0.63**
**Female age**	0.69	0.62–0.76	<0.001
**AMH**	1.04	0.93–1.15	0.45
**Endometrial thickness**	0.91	0.79–1.04	0.17
**Follicles ≥17 mm**	0.93	0.62–1.39	0.71

The **interaction is non-significant** → Protocol effect **does not differ** between PCOS and non-PCOS subgroups.
